# Exploring the Association between Parental Employment Status, Education Level, and Sensory Reactivity in Spanish Children Aged 3–7 Years: Findings from the InProS Study

**DOI:** 10.3390/children11070855

**Published:** 2024-07-14

**Authors:** Rocío Muñoz-Sánchez, Miriam Hurtado-Pomares, Iris Juárez-Leal, Jessica Piñero, Eva-María Navarrete-Muñoz, Desirée Valera-Gran

**Affiliations:** 1 Department of Surgery and Pathology, Miguel Hernández University, 03550 San Juan de Alicante, Spain; rocio.munozs@umh.es (R.M.-S.); mhurtado@umh.es (M.H.-P.); ijuarez@umh.es (I.J.-L.); dvalera@umh.es (D.V.-G.); 2 Occupational Therapy Research Group (InTeO, Investigación en Terapia Ocupacional), Miguel Hernández University, 03550 San Juan de Alicante, Spain; 3 Institute for Health and Biomedical Research of Alicante (ISABIAL, Instituto de Investigación Sanitaria y Biomédica de Alicante), 03010 Alicante, Spain; 4 Education Faculty, International University of La Rioja, 26006 Logroño, Spain; j.pinero@fundacionsaludinfantil.org; 5 Neurodevelopment Research Center, Fundación Salud Infantil, 03201 Elche, Spain; 6 Joint Research Unit UMH-Fisabio (STATSALUT), 03201 Elche, Spain

**Keywords:** sensory reactivity, employment status, education level, parental factors, children

## Abstract

This study explored the association between parental employment status and education level and the prevalence of sensory reactivity (SR) in population-based sample of school-aged children. SR was assessed in 495 children using the parent-reported Short Sensory Profile (SSP) questionnaire. Children with SR were identified based on probable or definitive differences in total SSP and subscales. Association between parental employment and education level were explored using multiple Poisson regression models with robust variance, adjusted for potential confounders. The main findings showed that a mother’s unemployment status was associated with higher prevalence of SR for the taste/smell sensitivity subscale (PR = 1.66, 95%CI: 1.08–2.56), and the low energy/weak (PR = 2.18, 95%CI: 1.31–3.49) subscale. A lower education level of a father was also associated with a higher prevalence of sensory problems on the tactile sensitivity subscale (PR_primary education_ = 2.68, 95%CI: 1.27–5.61; PR_secondary education_ = 1.96, 95%CI: 1.004–3.66) and the low energy/weak subscale (PR_secondary education_ = 1.95, 95%CI: 1.02–3.73). This study underscores the impact of parental employment and education on SR in school-aged children, offering insights for interventions and support systems aimed at improving their sensory functioning and overall well-being.

## 1. Introduction

Sensory processing (SP) refers to the neurobiological capacity to register, analyse, organise, and integrate internal and external information received by the senses (visual, auditory, tactile, olfactory, gustatory, proprioceptive, vestibular, and interoceptive stimuli) to generate adaptative responses to the demands of our own body and the environment [1]. Difficulties in SP can manifest as challenges in processing, responding to, and/or organising sensory information, which may lead to different health issues, including sensory reactivity (SR) [2]. Investigations concerning children with SR have revealed that they often encounter difficulties related to emotional regulation, motor performance, social interaction, and functional performance in activities of daily living at home, at school, and/or in the community [3,4,5].

Research in the field of SR has primarily concentrated on children with developmental disorders and/or other specific medical conditions, such as autism spectrum disorder [6], attention-deficit/hyperactivity disorder, or prematurity [7]. This focus is attributed to the notably high prevalence of reactivity issues in these populations, which can be observed in a substantial proportion of cases, ranging from 44% to 88% [8]. However, studies performed in children aged 3 to 11 years without these conditions have shown that the prevalence of SR can vary widely, ranging from approximately 5–10% to 50–55% [9,10,11,12].

While these studies provide valuable insights, there is a significant gap in understanding the environmental factors associated with SR in children without medical conditions. Epidemiological evidence in this area is practically non-existent. To date, only a few studies have explored the association between different lifestyle factors, such as sleep, body mass index, quality of diet (measured by adherence to the Mediterranean diet), and SR in childhood [12,13,14]. However, the association between contextual factors, including parental socio-demographic characteristics, and prevalence of SR in children remains unknown.

Existing studies have shown that parental employment and educational level can significantly impact children’s physical, cognitive, and emotional development [15,16,17,18,19,20,21,22]. For instance, higher parental education has been associated with better neurocognitive development and deductive reasoning in children [15], while socio-economic inequalities have been linked to disparities in adolescent health outcomes [16]. Research indicates that parental social class and employment status can impact child cognitive development. Specifically, maternal education, age, and intelligence, as well as paternal social class and the child’s age and sex, were found to be significantly associated with cognitive development [17]. Additionally, parental education levels have a strong influence on parent-reported child mental health, which is more significant than the effects of other variables such as family income and social class [21]. While some research has independently linked specific parental socio-demographic factors and SR to negative health outcomes in children, no prior studies have specifically explored the association between these factors. Therefore, the present study aimed to examine the association between parental employment status and education level and SR in children aged 3–7 years from the Spanish population.

## 2. Materials and Methods

### 2.1. Study Design and Participants

This study is part of the InProS project (Infancia y Procesamiento Sensorial: (https://inteo.edu.umh.es/inpros/, accessed on 1 June 2024), a cross-sectional population-based study aimed at assessing the prevalence of SR and examining its association with socio-demographic and lifestyle factors in children aged 3 to 7 years. More information on the study design and variables can be found in the study protocol [23]. The recruitment of participants took place from February to May 2016 across 21 schools randomly selected in the province of Alicante, Spain.

A total of approximately1700 children were enrolled in this study after obtaining permission of the principal of each school. The teachers provided an envelope to the children that included the following documents: a sheet with details about the project, informed consent, a booklet comprising several questionnaires to be completed by parents/guardians, and guidelines on how to fill them out. Following a period of approximately two to three weeks, the children returned the provided materials to their respective teachers, who would then deliver them to the research staff. Of the total number of children who were invited to participate, 620 children returned the study documentation, yielding an approximate response rate of 37%. The current study included a total of 495 pairs of parents and children who had complete data on all the study variables.

All participants submitted informed consent signed by parents/legal guardians and received no compensation for their involvement in this study. The InProS study has the approval of the Research Ethics Committee of Miguel Hernández University (DPC.ASP.02.16; DPC.ENM.080323) and was performed in accordance with the Declaration of Helsinki and current Spanish legislation on data protection (Ley Orgánica 3/2018, de 5 de diciembre, de Protección de Datos Personales y garantía de los derechos digitales).

### 2.2. Measures

#### 2.2.1. Sensory Reactivity

SR was assessed using the Short Sensory Profile (SSP), a screening tool designed by W. Dunn [24] and cross-culturally adapted for use with Spanish children [25]. The SSP is a parent/guardian-reported questionnaire consisting of 38 items, subdivided into seven subscales assessing the different sensory systems. Each item in the scale is assigned a score ranging from 1 (always) to 5 (never). The total score is obtained by adding the scores of all 38 items on the scale. The SSP also allows scoring each subscale by summing up the corresponding items within each subscale. Based on the classification proposed by Dunn [24], the scores obtained can classify children into three SP profiles, both globally and for each of the subscales: typical functioning, probable difference, and definitive difference. For the present study, children exhibiting SR were identified based on being classified as having a probable difference or definitive difference on the total SSP score and each subscale or scoring below the following thresholds: total SSP (<155 points), tactile sensitivity (<30 points), taste/smell sensitivity (<15 points), movement sensitivity (<13 points), under-responsive/seeks sensation (<27 points), auditory filtering (<23 points), low energy/weak (<27 points) and visual/auditory sensitivity (<19 points).

#### 2.2.2. Parental Employment and Education Level

Socio-demographic information was self-reported by parents through an ad hoc questionnaire based on previous studies [26]. Parental employment status was collected using the question “What is your current employment status?” with the following response options: worker, unemployed, student, on leave, homeworker, and others. Regarding education level, we used the question “What level of education have you completed?” with the answer options: illiterate, no studies or incomplete primary studies, primary studies, secondary studies, university studies, and others. To perform the analysis, education level was recategorized as primary education (illiterate, no studies or incomplete primary studies, and primary studies), secondary education, and higher education (university studies, others). A dichotomous variable was created for employment status: employed (worker) and unemployed (unemployed, student, on leave, homeworker, and others).

#### 2.2.3. Covariates

To mitigate potential confounding factors, we considered additional socio-demographic and lifestyle information concerning both parents and their children. For maternal and paternal characteristics, main models included age (in years), country of birth (Spain, other country), smoking (yes, no), and marital status (married/partnership, single/widowed/divorced). We also considered child-specific covariates, including age (in years), sex (female, male), adherence to the Mediterranean diet measured with the Mediterranean Diet Quality Index (KIDMED) categorised as low, medium, high [27], and sleep quality (good, poor) evaluated using the Spanish version of the Paediatric Sleep Questionnaire (PSQ) [28].

#### 2.2.4. Statistical Analysis

Statistical analysis was performed using the statistical software R version R 4.0.2. (http://www.r-project.org). We conducted two-tailed tests with a significance level set at 0.05. Normality of continuous variables was assessed using the Kolmogorov–Smirnov test with Lilliefors correction.

A descriptive analysis of the socio-demographic and lifestyle variables of the parents and their children was conducted according to the global SR of the children (SSP ≥ 155 vs. SSP < 155) using frequencies (n) and percentages (%) for categorical variables and median and interquartile range (IQR) for quantitative variables. To compare differences between socio-demographic and lifestyle variables, we utilised the chi-squared (χ^2^) or Fisher’s exact test for categorical variables and the Mann–Whitney U test for continuous variables.

The association between parental employment status and education level and the prevalence of SR was explored by estimating prevalence ratios (PRs) using multiple Poisson regression models with robust variance estimation based on Huber’s sandwich method. The models were adjusted for variables that showed a *p*-value < 0.20 in the bivariate analysis and/or caused > 10% changes in the association’s magnitude when included.

## 3. Results

In the present study, the prevalence of global SR (SSP < 155 points) of the children was 28.3%, with the prevalence for each SSP subscale being 11.1% (tactile sensitivity < 30), 14.8% (taste/smell sensitivity < 15), 22.2% (movement sensitivity < 13), 48.1% (under-responsive/seeks sensation < 27), 41.2% (auditory filtering < 23), 12.3% (low energy/weak < 26), and 26.1% (visual/auditory sensitivity < 19).

The socio-demographic and lifestyle characteristics of the InProS study participants according to the children’s global SR are shown in Table 1. Regarding parental characteristics, the median age was 38 (IQR: 35–41) for mothers and 40 (IQR: 37–43) for fathers. Over 15% of parents were born in a country other than Spain, and approximately 80% were married or had a partner. Regarding the characteristics of the children, there was a proportionate representation of both sexes (male = 50.5%; female = 49.5%), and the median age was five years (IQR: 4–6). Almost half of the children had low adherence to the Mediterranean diet and 8.1% had poor sleep quality. Considering the sensory profile, results showed that children with SR (SSP < 155) compared to children without SR (SSP ≥ 155), were more likely to have younger (37 vs. 38) mothers and both parents born in a foreign country (mothers = 27.9% vs. 11.5%; fathers = 28.6% vs. 13.0%). We also observed a statistically significantly higher prevalence of SR in boys than in girls (61.4% vs. 38.6%). Compared to children classified without SR, we observed children with SR were high in proportion with low adherence to the Mediterranean diet (55.7% vs. 44.3%) and poor sleep quality (20.3% vs. 3.2%).

Table 2 and Table 3 provide a comparison of the employment status and education level of both parents, with Table 2 focusing on the mother and Table 3 on the father, based on the presence or absence of SR in the children. The prevalence of unemployment was 29.7% in the mothers and 10.3% in the fathers. Children with non-working mothers had a higher prevalence of SR in all SSP subscales, except for the tactile sensitivity (15.0% vs. 9.5%, *p* = 0.085), under-responsive/seeks sensation (53.7% vs. 45.7%, *p* = 0.115), and auditory filtering subscales (40.1% vs. 41.7%, *p* = 0.765). Regarding the fathers, no statistically significant differences were observed in children’s SR rates between fathers who were employed and those who were unemployed.

Overall, the results showed no significant differences in the presence of SR in children regarding the different levels of maternal education. However, an exception was observed in the mothers with primary education, who had a higher rate of children with SR on the under-responsive/seeks sensation subscale (58.6%) compared with mothers with secondary (46.8%) and higher (43.6%) education levels. In relation to the father’s formal education, it was observed that children whose fathers had secondary education showed a higher prevalence of SR on the total SSP (33.5% vs. 30.2% (primary education) and 21.1% (higher education), *p* = 0.032), taste/smell sensitivity (21.2% vs. 11.3% (primary education) and 11.4% (higher education), *p* = 0.014), and low energy/weak (18.8% vs. 10.1% (primary education) and 7.8% (higher education), *p* = 0.005) scales.

Table 4 and Table 5 display the results of the association between parental employment status and education level and SR for the total score and subscales of the SSP. In relation to the mothers (Table 4), children whose mothers were unemployed were more likely to have SR on the taste/smell sensitivity subscale (PR = 1.66, 95%CI: 1.08–2.56) and low energy/weak subscale (PR = 2.18, 95%CI: 1.36–3.49) of the SSP compared to children whose mothers were employed. Regarding the maternal education level, it was observed that children whose mothers had primary or secondary education were less likely to have SR on the tactile sensitivity subscale (PR_primary education_ = 0.35, 95%CI: 0.15–0.82; PR_secondary education_ = 0.53, 95%CI: 0.31–0.92, respectively) compared to children whose mothers had a higher education level.

No significant association was observed between paternal unemployment and SR in their children (Table 5). However, a statistically significant association was observed between the father’s education level and children’s SR in certain subscales of the SSP. Compared to children whose fathers had a higher education, children whose fathers had primary or secondary education were more likely to have SR on the tactile sensitivity subscale (PR_primary education_ = 2.68, 95%CI: 1.27–5.61; PR_secondary education_ = 1.926, 95%CI: 1.04–3.66, respectively). Similarly, children whose fathers had secondary education were more likely to have SR on the low energy/weak subscale (PR = 1.95, 95%CI: 1.02–3.73) compared to those whose fathers had a higher education level.

## 4. Discussion

The present study found that approximately one-third of the Spanish school-aged children included had SR based on the total SSP score. The main results showed that parental unemployment and lower levels of education are contextual factors potentially associated with a higher prevalence of SR in children. Specifically, we observed that maternal unemployment status was associated with a higher prevalence of SR on the taste/smell sensitivity and the low energy/weak subscales of the SSP. Moreover, a lower paternal education level was associated with a higher prevalence of sensory problems on the tactile sensitivity and low energy/weak sensation subscales. While the exact causes of these associations remain unknown, it is plausible to consider that experiencing unemployment and having a lower parental education level may increase the likelihood of growing up in an unfavourable environment, which could negatively impact children’s sensory processing development [29].

Our results are consistent with previous studies that have shown the link between lower socio-economic status variables and SR as measured by the SSP. For instance, Gouze et al. (2009) conducted a longitudinal study using a similar epidemiological approach with a representative community sample of 4-year-old children. Their findings showed that lower socio-economic status was associated with sensory regulation dysfunction, as measured by the total SSP score, reflecting global SR [30]. Similarly, Gunn et al. (2009) found a correlation between SR in children and low family socio-economic status [31]. Another study involving Puerto Rican pre-schoolers in 2012 revealed that a lower level of parental education was linked to a higher prevalence of SR on the low energy/weak subscale [32].

Interestingly, our findings not only revealed an association between lower parental education level or unemployment status and higher rates of SR problems but also a protective effect of mothers with lower education levels (primary education/secondary education) on the prevalence of tactile SR in their children. This unexpected finding warrants further investigation and may suggest that mothers with lower education levels might have limited awareness of the presence of SR in their children, potentially leading to under-reporting. It is possible that less educated mothers may possess less knowledge about child development, which might hinder their ability to recognize certain deficits in their children.

Nevertheless, by considering these findings in conjunction with previous research, our study contributes to the growing body of evidence linking parental contextual factors and child SR issues. In addition to supporting these earlier findings, it should be noted that the present study complements the epidemiological approach by broadening the scope of research on SR. This expansion occurs on multiple fronts. First, we conducted a comprehensive evaluation of sensory domains, quantifying the effect on all SSP subscales. Second, we examined the association with socio-economic status, analysing socio-demographic characteristics, such as employment status and education level separately. This approach helps to disentangle the individual contributions of these factors and provides a clearer picture of their influence on child SR. Finally, our research delved into potential differences in the influence of parental context on the child’s SR by specifically examining the role of both mothers and fathers. Through this comprehensive approach, our study provides a more nuanced understanding of the complexities underlying the relationship between socio-economic factors, such as parental employment and education level, and SR in children.

Existing research has shown a link between parental socio-demographic characteristics, such as employment status and education, and overall child development, particularly at neurodevelopmental level [33]. Specifically, lower socio-economic status of parents, including their employment status, education level, and income, has been related to alterations in the child’s brain structure. Studies have showed reduced grey matter volume in specific brain regions, including the bilateral hippocampus, medial temporal gyrus, left fusiform gyrus, and right inferior occipitotemporal gyrus, which are known to be critical for cognitive development [34]. Consistent with this evidence, extensive research suggests that the socio-economic context in which children grow up has a notable impact on their physical, neurological, and cognitive development. These developmental effects in turn have implications for their social participation and educational attainment [15,16,17,19,21,22,35]. However, the association between sensory aspects of neuropsychological development, such as difficulties with sensory regulation and processing, and socio-economic factors has received limited investigation. By examining these less explored sensory aspects, we can gain a more comprehensive understanding of the impact of socio-economic factors on the overall development of children. This knowledge can be helpful in informing targeted interventions and support for children experiencing sensory challenges in these contexts.

This study has certain limitations that warrant acknowledgement. The cross-sectional design of this study does not allow for a cause–effect association. However, in the case of parental determinants such as education level or unemployment potentially preceding a child’s SR, it can be argued that the temporal relationship might be plausible. Given that the study data relied on parental reports, the potential for classification bias cannot be ruled out; nonetheless, any inaccuracies should not be differential. The SSP is a well-known, accurate, and valid screening measure for identifying SR issues. However, to provide a more holistic understanding of sensory domains, it would be necessary that future studies consider including performance tests or direct measures of child behaviour. Regarding the socio-demographic variables, an important limitation is that we did not specifically ask about parental socio-economic status. Socio-economic status encompasses various factors, including parental education, employment, and income [36]. However, the questions on employment status and education level included in the study are based on questionnaires used in other high-quality studies conducted on child health [26]. These two parameters are recognised as key components in forming the multidimensional construct of socio-economic status [30,35]. In addition, to measure SR, we used the Spanish version of SSP, which was adapted to the present study population. Although the adaptation of SSP ensured its suitability for this context, it is important to note that it was not specifically validated for this study. However, the adaptation enhances the accuracy of the measure within the study. While efforts were made to account for many potential confounders in the main analyses, it is important to acknowledge that residual confounding or bias due to uncollected information cannot be ignored. Finally, the random selection of centres for recruitment of participants ensured the population representativeness of the sample. This enhances the potential extrapolation of the results to the general population.

## 5. Conclusions

To our knowledge, this study represents a pioneering effort within the Spanish population to explore the association between parental employment status and education level and the prevalence of SR in children aged 3–7 years. The findings reveal compelling insights, showing that maternal unemployment status may be associated with higher presence of SR on the taste/smell and low energy/weak sensitivity subscales of the SSP. In addition, lower paternal education level was also associated with higher prevalence of RS on the tactile sensitivity and low energy/weak subscales. These results hold significant implications for children’s development, as SR can potentially hinder their appropriate sensory processing and consequently impact their occupational performance and that of their families. Given the scarcity of existing research in this area, this study underscores the critical need for population studies with a longitudinal design to confirm these findings and to provide further scientific evidence on the association between contextual factors and sensory problems in childhood. By addressing this research gap, future studies can contribute to enhancing the evidence base for effective sensory-focused interventions and family-centred approaches.

## Figures and Tables

**Table 1:** General characteristics of mothers, fathers, and children participating in the InProS project according to total score on the Short Sensory Profile (SSP) (n = 495).

Study Variables	Total	Sensory Reactivity		*p* Value *
		SSP ≥ 155	SSP < 155	
	n (%)	n (%)	n (%)	
**Maternal Characteristics**	495 (100)	355 (71.7)	140 (28.3)	
Age (years), median (IR)	38 (35–41)	38 (35–41)	37 (33–41)	0.013
Country of birth				< 0.001
Spain	415 (83.8)	314 (88.5)	101 (72.1)	
Others	90 (16.2)	41 (11.5)	39 (27.9)	
Marital status				0.215
Married/partnership	394 (79.6)	288 (81.1)	106 (75.7)	
Single/widowed/divorced	101 (20.4)	67 (18.9)	34 (24.3)	
**Paternal Characteristics**				
Age (years), median (IR)	40 (37–43)	40 (37–43)	40 (36–43)	0.221
Country of birth				< 0.001
Spain	409 (82.6)	309 (87.0)	100 (71.4)	
Others	86 (17.4)	46 (13.0)	40 (28.6)	
Marital status				0.218
Married/partnership	393 (79.6)	287 (80.8)	106 (75.7)	
Single/widowed/divorced	101 (20.4)	68 (19.2)	34 (24.3)	
**Child Characteristics**				
Sex				0.003
Male	250 (50.5)	164 (46.2)	86 (61.4)	
Female	245 (49.5)	191 (53.8)	54 (38.6)	
Age (years), median (IR)	5 (4–6)	5 (4–6)	5 (4–6)	0.483
Adherence to MD				0.006
Low	227 (45.9)	149 (42.0)	78 (55.7)	
Medium–High	268 (54.1)	206 (58.0)	62 (44.3)	
Sleep Quality				<0.001
Good	455 (91.9)	345 (97.2)	103 (78.6)	
Poor	40 (8.1)	10 (2.8)	30 (21.4)	

Abbreviations: InProS, Infancia y Procesamiento Sensorial; MD, Mediterranean diet; IR, interquartile range. * *p*-value was calculated using the Fisher’s exact test for categorical variables and by Mann–Whitney U test for continuous variables.

**Table 2:** Comparison of mothers’ and fathers’ employment status (unemployed or employed) according to SR (total SSP scores and subscales) in children aged 3–7 years from the InProS Project (n = 495).

Short Sensory Profile	Total	Mother Employment		*p* Value *	Father Employment		*p* Value *
		Unemployed	Employed		Unemployed	Employed	
	n (%)	n (%)	n (%)		n (%)	n (%)	
	495 (100)	147 (29.7)	348 (70.3)		51 (10.3)	444 (89.7)	
**Total score** (Items 1–38)				0.002			0.870
No SR (155–190 points)	355 (71.7)	91 (61.9)	264 (75.9)		36 (70.6)	319 (71.8)	
SR (38–154 points)	140 (28.3)	56 (38.1)	84 (24.1)		15 (29.4)	125 (28.2)	
**Tactile sensitivity** (Items 1–7)				0.085			0.153
No SR (30–35 points)	440 (88.9)	125 (85.0)	315 (90.5)		42 (82.4)	398 (89.6)	
SR (7–29 points)	55 (11.1)	22 (15.0)	33 (9.5)		9 (17.6)	46 (10.4)	
**Taste/smell sensitivity** (Items 5–11)				<0.001			0.677
No SR (15–20 points)	422 (85.2)	112 (76.2)	310 (89.1)		45 (88.2)	377 (84.9)	
SR (4–14 points)	73 (14.8)	35 (23.8)	38 (10.9)		6 (11.8)	67 (15.1)	
**Movement sensitivity** (Items 12–14)				0.002			0.391
No SR (13–15 points)	385 (77.8)	101 (68.7)	284 (81.6)		39 (76.5)	346 (77.9)	
SR (3–12 points)	110 (22.2)	46 (31.3)	64 (18.4)		12 (23.5)	98 (22.1)	
**Under-responsive/seeks sensation** (Items 15–21)				0.115			0.374
No SR (27–35 points)	257 (51.9)	68 (46.3)	189 (53.7)		23 (45.1)	234 (52.7)	
SR (7–26 points)	238 (48.1)	79 (53.7)	159 (45.7)		28 (54.9)	210 (47.3)	
**Auditory filtering** (Items 22–27)				0.765			1.00
No SR (23–30 points)	291 (58.8)	88 (59.9)	203 (58.3)		30 (58.8)	261 (58.8)	
SR (22–6 points)	204 (41.2)	59 (40.1)	145 (41.7)		21 (41.2)	183 (41.2)	
**Low energy/weak** (Items 26–33)				<0.001			0.257
No SR (26–30 points)	534 (87.7)	114 (77.6)	320 (92.0)		42 (82.4)	392 (88.3)	
SR (6–25 points)	61 (12.3)	33 (22.4)	28 (8.0)		9 (17.6)	52 (11.7)	
**Visual/auditory sensitivity** (Items 34–38)				<0.001			0.179
No SR (19–25 points)	366 (73.9)	112 (76.2)	310 (89.1)		42 (82.4)	324 (73.0)	
SR (5–18 points)	129 (26.1)	35 (23.8)	38 (10.9)		9 (17.6)	120 (27.0)	

Abbreviations: SSP, Short Sensory Profile; InProS, Infancia y Procesamiento Sensorial; SR, sensory reactivity. * *p*-value was calculated using Fisher’s exact test.

**Table 3:** Comparison of mothers’ and fathers’ education level (primary, secondary, or higher education) according to SR (total SSP scores and subscales) in children aged 3 to 7 years from the InProS Project (n = 495).

Short Sensory Profile	Total	Mother’s Level of Education			*p* Value *	Father’s Level of Education			*p* Value *
		Primary	Secondary	Higher		Primary	Secondary	Higher	
	n (%)	n (%)	n (%)	n (%)		n (%)	n (%)	n (%)	
		111 (22.4)	173 (35.0)	211 (42.6)		159 (32.2)	170 (34.3)	166 (33.5)	
**Total score** (Items 1–38)					0.111				0.032
No SR (155–190 points)	355 (71.7)	73 (65.8)	121 (69.9)	161 (76.3)		111 (69.8)	113 (66.5)	131 (78.9)	
SR (38–154 points)	140 (28.3)	38 (34.2)	52 (30.1)	50 (23.7)		48 (30.2)	57 (33.5)	35 (21.1)	
**Tactile sensitivity** (Items 1–7)					0.973				0.143
No SR (30–35 points)	440 (88.9)	99 (89.2)	153 (88.4)	188 (89.1)		139 (87.4)	147 (86.5)	154 (92.8)	
SR (7–29 points)	55 (11.1)	12 (10.8)	20 (11.6)	23 (10.9)		20 (12.6)	23 (13.5)	12 (7.2)	
**Taste/smell sensitivity** (Items 5–11)					0.362				0.014
No SR (15–20 points)	422 (85.2)	91 (82.0)	146 (84.4)	285 (87.7)		141 (88.7)	134 (78.8)	147 (88.6)	
SR (4–14 points)	73 (14.8)	20 (18.0)	27 (15.6)	26 (12.3)		18 (11.3)	36 (21.2)	19 (11.4)	
**Movement sensitivity** (Items 12–14)					0.124				0.224
No SR (13–15 points)	385 (22.2)	85 (76.6)	127 (73.4)	273 (82.0)		123 (77.4)	126 (74.1)	136 (81.9)	
SR (3–12 points)	110 (77.8)	26 (23.4)	46 (26.6)	38 (18.0)		36 (22.6)	44 (25.9)	30 (18.1)	
**Under-responsive/seeks sensation** (Items 15–21)					0.035				0.521
No SR (27–35 points)	257 (51.9)	46 (41.4)	92 (53.2)	119 (56.4)		87 (50.9)	88 (49.2)	96 (54.9)	
SR (7–26 points)	238 (48.1)	65 (58.6)	81 (46.8)	92 (43.6)		84 (49.1)	91 (50.8)	79 (45.1)	
**Auditory filtering** (Items 22–27)					0.727				0.559
No SR (23–30 points)	291 (58.8)	65 (58.6)	98 (56.6)	128 (60.7)		92 (57.9)	96 (56.5)	103 (62.0)	
SR (22–6 points)	204 (41.2)	46 (41.4)	75 (43.4)	83 (39.3)		67 (42.1)	74 (43.5)	63 (38.0)	
**Low energy/weak** (Items 26–33)					0.252				0.005
No SR (26–30 points)	534 (87.7)	95 (85.6)	148 (85.5)	191 (90.5)		143 (89.9)	138 (82.2)	153 (92.2)	
SR (6–25 points)	61 (12.3)	16 (14.4)	25 (14.5)	20 (9.5)		16 (10.1)	32 (18.8)	13 (7.8)	
**Visual/auditory sensitivity** (Items 34–38)					0.064				0.372
No SR (19–25 points)	366 (73.9)	86 (77.5)	117 (67.6)	163 (77.3)		116 (73.0)	121 (71.2)	129 (77.7)	
SR (5–18 points)	129 (26.1)	25 (22.5)	56 (32.4)	48 (22.7)		43 (27.0)	49 (28.8)	37 (22.3)	

Abbreviations: SSP, Short Sensory Profile; InProS, Infancia y Procesamiento Sensorial; SR, sensory reactivity. * *p*-value was calculated using the chi-squared test.

**Table 4:** Association between mother’s employment status and education level and the prevalence of SR in children aged 3–7 years in the InProS project (n = 495).

Short Sensory Profile	Prevalence	Mother’s Employment Status and Education Level											
		Unemployed				Primary Education				Secondary Education			
		n Cases (%)	PR	95% CI	*p*	n Cases (%)	PR	95% CI	*p* Value	n Cases (%)	PR	95% CI	*p*
Total score (SSP < 155 points)	28.3	56 (38.1)	1.32	0.99–1.78	0.062	38 (34.2)	0.85	0.56–1.29	0.447	52 (30.1)	0.85	0.58–1.22	0.369
Tactile sensitivity (SSP < 30 points)	11.1	22 (15.0)	1.10	0.63–1.95	0.732	12 (10.8)	0.35	0.15–0.82	0.015	20 (11.6)	0.53	0.31–0.92	0.024
Taste/smell sensitivity (SSP < 15 points)	14.8	35 (23.8)	1.66	1.08–2.56	0.022	20 (18.0)	0.86	0.47–1.58	0.630	27 (15.6)	0.79	0.46–1.34	0.372
Movement sensitivity (SSP < 13 points)	77.8	46 (31.3)	1.34	0.94–1.90	0.098	26 (23.4)	0.92	0.53–1.60	0.775	46 (26.6)	1.14	0.74–1.75	0.559
Under-responsive/seeks sensation (SSP < 26 points)	48.1	79 (53.7)	1.04	0.86–1.27	0.682	65 (58.6)	1.13	0.86–1.48	0.368	81 (46.8)	0.95	0.75–1.20	0.688
Auditory filtering (SSP < 23 points)	41.2	59 (40.1)	0.86	0.68–1.09	0.238	46 (41.4)	0.83	0.59–1.15	0.270	75 (43.4)	0.95	0.72–1.25	0.694
Low energy/weak (SSP < 27 points)	12.3	33 (22.4)	2.18	1.36–3.49	0.001	16 (14.4)	0.86	0.43–1.71	0.678	25 (14.5)	0.90	0.51–1.60	0.728
Visual/auditory sensitivity (SSP < 19 points)	26.1	35 (23.8)	1.03	0.75–1.41	0.841	25 (22.5)	0.76	0.46–1.23	0.264	56 (32.4)	1.14	0.78–1.65	0.501

Abbreviations: SSP, Short Sensory Profile; InProS, Infancia y Procesamiento Sensorial; SR, sensory reactivity; PR, prevalence ratio; CI, confidence interval. Models were adjusted for mother’s country of birth (Spain or other), mother’s age (continuous), child´s age (continuous), child´s sex (male or female), child’s sleep quality (good or bad), child´s adherence to the Mediterranean diet (continuous), father’s employment status (employed or unemployed), father´s education level (primary, secondary or higher education), and father´s country of birth (Spain or other).

**Table 5:** Association between father’s employment status and education level and the prevalence of SR in children aged 3–7 years in the InProS project (n = 495).

Short Sensory Profile	Prevalence	Father’s Employment Status and Education Level											
		Unemployed				Primary Education				Secondary Education			
		n Cases (%)	PR	95% CI	*p*	n Cases (%)	PR	95% CI	*p* Value	n Cases (%)	PR	95% CI	*p*
Total score (SSP < 155 points)	28.3	15 (29.4)	0.84	0.54–1.32	0.460	48 (30.2)	1.35	0.88–2.08	0.166	57 (33.5)	1.40	0.97–2.04	0.074
Tactile sensitivity (SSP < 30 points)	11.1	9 (17.6)	1.25	0.67–2.36	0.487	20 (12.6)	2.68	1.27–5.61	0.008	23 (13.5)	1.96	1.04–3.66	0.034
Taste/smell sensitivity (SSP < 15 points)	14.8	6 (11.8)	0.59	0.26–1.35	0.209	18 (11.3)	0.94	0.47–1.85	0.847	36 (21.2)	1.46	0.86–2.48	0.156
Movement sensitivity (SSP < 13 points)	77.8	12 (23.5)	0.80	0.49–1.31	0.370	36 (22.6)	1.22	0.73–2.03	0.446	44 (25.9)	1.26	0.80–1.98	0.306
Under-responsive/seeks sensation (SSP < 26 points)	48.1	28 (54.9)	1.04	0.81–1.34	0.742	84 (49.1)	0.97	0.74–1.28	0.798	91 (50.8)	1.03	0.81–1.30	0.777
Auditory filtering (SSP < 23 points)	41.2	21 (41.2)	0.96	0.68–1.33	0.790	67 (42.1)	1.13	0.82–1.54	0.448	74 (43.5)	1.10	0.84–1.45	0.492
Low energy/weak (SSP< 27 points)	12.3	9 (17.6)	1.17	0.63–2.20	0.626	16 (10.1)	1.18	0.56–2.55	0.663	32 (18.8)	1.95	1.02–3.73	0.039
Visual/auditory sensitivity (SSP< 19 points)	26.1	9 (17.6)	0.57	0.31–1.04	0.068	43 (27.0)	1.34	0.87–2.06	0.192	49 (28.8)	1.21	0.82–1.79	0.336

Abbreviations: SSP, Short Sensory Profile; InProS, Infancia y Procesamiento Sensorial; SR, sensory reactivity; PR, prevalence ratio; CI, confidence interval. Models were adjusted for mother’s country of birth (Spain or other), mother’s age (continuous), child´s age (continuous), child´s sex (male or female), child’s sleep quality (good or bad), child´s adherence to the Mediterranean diet (continuous), father’s employment status (employed or unemployed), father´s education level (primary, secondary or higher education), and father´s country of birth (Spain or other).

## Data Availability

There are restrictions on the availability of data for this study because of ethical and legal restrictions implemented by the Ethics Committee of Miguel Hernández University. In the informed consent forms for participants, the authors guaranteed the confidentiality of collected personal information. However, all data used in the submitted manuscript may be available to interested researchers upon request to the corresponding author, Eva-María Navarrete-Muñoz, who is the guarantor of the study database. Requests will be reviewed by the research team and will require a data transfer agreement. Interested researchers should contact Eva-María Navarrete-Muñoz (enavarrete@umh.es).

## References

[1] Chien C.-W., Rodger S., Copley J., Branjerdporn G., Taggart C. (2016). Sensory Processing and Its Relationship with Children’s Daily Life Participation. Phys. Occup. Ther. Pediatr..

[2] Miller L.J., Anzalone M.E., Lane S.J., Cermak S.A., Osten E.T. (2007). Concept Evolution in Sensory Integration: A Proposed Nosology for Diagnosis. Am. J. Occup. Ther. Off. Publ. Am. Occup. Ther. Assoc..

[3] Dunn W., Little L., Dean E., Robertson S., Evans B. (2016). The State of the Science on Sensory Factors and Their Impact on Daily Life for Children: A Scoping Review. OTJR Occup. Particip. Health.

[4] Miller L.J., Nielsen D.M., Schoen S.A., Brett-Green B.A. (2009). Perspectives on Sensory Processing Disorder: A Call for Translational Research. Front. Integr. Neurosci..

[5] Miller L.J., Schoen S.A., Mulligan S., Sullivan J. (2017). Identification of Sensory Processing and Integration Symptom Clusters: A Preliminary Study. Occup. Ther. Int..

[6] Tomchek S.D., Dunn W. (2007). Sensory Processing in Children with and without Autism: A Comparative Study Using the Short Sensory Profile. Am. J. Occup. Ther. Off. Publ. Am. Occup. Ther. Assoc..

[7] Bröring T., Königs M., Oostrom K.J., Lafeber H.N., Brugman A., Oosterlaan J. (2018). Sensory Processing Difficulties in School-Age Children Born Very Preterm: An Exploratory Study. Early Hum. Dev..

[8] Critz C., Blake K., Nogueira E. (2015). Sensory Processing Challenges in Children. J. Nurse Pract..

[9] Delgado-Lobete L., Montes-Montes R., Rodríguez Seoane S. (2016). Prevalencia de trastorno del procesamiento sensorial en niños españoles. Resultados preliminares y comparación entre herramientas de diagnóstico. Rev. Electrónica Ter. Ocup. Galicia TOG.

[10] Jorquera-Cabrera S., Romero-Ayuso D., Rodriguez-Gil G., Triviño-Juárez J.-M. (2017). Assessment of Sensory Processing Characteristics in Children between 3 and 11 Years Old: A Systematic Review. Front. Pediatr..

[11] Kong M., Moreno M.A. (2018). Sensory Processing in Children. JAMA Pediatr..

[12] Navarrete-Muñoz E.-M., Fernández-Pires P., Mubarak-García C., Espinosa-Sempere C., Peral-Gómez P., Juárez-Leal I., Sánchez-Pérez A., Pérez-Vázquez M.-T., Hurtado-Pomares M., Valera-Gran D. (2020). Association between Body Mass Index and Sensory Processing in Childhood: InProS Study. Nutrients.

[13] Fernández-Pires P., Valera-Gran D., Hurtado-Pomares M., Espinosa-Sempere C., Sánchez-Pérez A., Juárez-Leal I., Ruiz-Carbonell M.-P., Peral-Gómez P., Campos-Sánchez I., Pérez-Vázquez M.-T. (2021). Sleep Duration and Quality and Sensory Reactivity in School-Aged Children: The Spanish Cross-Sectional InProS Study. Front. Pediatr..

[14] Navarrete-Muñoz E.-M., Fernández-Pires P., Navarro-Amat S., Hurtado-Pomares M., Peral-Gómez P., Juárez-Leal I., Espinosa-Sempere C., Sánchez-Pérez A., Valera-Gran D. (2019). Association between Adherence to the Antioxidant-Rich Mediterranean Diet and Sensory Processing Profile in School-Aged Children: The Spanish Cross-Sectional InProS Project. Nutrients.

[15] Demir-Lira Ö.E., Prado J., Booth J.R. (2021). Neurocognitive Basis of Deductive Reasoning in Children Varies with Parental Education. Hum. Brain Mapp..

[16] Elgar F.J., Pförtner T.-K., Moor I., De Clercq B., Stevens G.W.J.M., Currie C. (2015). Socioeconomic Inequalities in Adolescent Health 2002–2010: A Time-Series Analysis of 34 Countries Participating in the Health Behaviour in School-Aged Children Study. Lancet Lond. Engl..

[17] González L., Cortés-Sancho R., Murcia M., Ballester F., Rebagliato M., Rodríguez-Bernal C.L. (2020). The Role of Parental Social Class, Education and Unemployment on Child Cognitive Development. Gac. Sanit..

[18] Hormiga-Sánchez C.M., Alzate-Posada M.L., Borrell C., Palència L., Rodríguez-Villamizar L.A., Otero-Wandurraga J.A. (2016). Occupation-, transportation- and leisure-related physical activity: Gender inequalities in Santander, Colombia. Rev. Salud Publica Bogota Colomb..

[19] Raizada R.D.S., Kishiyama M.M. (2010). Effects of Socioeconomic Status on Brain Development, and How Cognitive Neuroscience May Contribute to Levelling the Playing Field. Front. Hum. Neurosci..

[20] Salimiha A., Perales F., Baxter J. (2018). Maternal Employment and Children’s Socio-Emotional Outcomes: An Australian Longitudinal Study. Int. J. Public Health.

[21] Sonego M., Llácer A., Galán I., Simón F. (2013). The Influence of Parental Education on Child Mental Health in Spain. Qual. Life Res. Int. J. Qual. Life Asp. Treat. Care Rehabil..

[22] Ursache A., Noble K.G. (2016). Neurocognitive Development in Socioeconomic Context: Multiple Mechanisms and Implications for Measuring Socioeconomic Status. Psychophysiology.

[23] Fernández-Pires P., Valera-Gran D., Sánchez-Pérez A., Hurtado-Pomares M., Peral-Gómez P., Espinosa-Sempere C., Juárez-Leal I., Navarrete-Muñoz E.-M. (2020). The Infancia y Procesamiento Sensorial (InProS-Childhood and Sensory Processing) Project: Study Protocol for a Cross-Sectional Analysis of Parental and Children’s Sociodemographic and Lifestyle Features and Children’s Sensory Processing. Int. J. Environ. Res. Public. Health.

[24] Dunn W. (1994). Performance of Typical Children on the Sensory Profile: An Item Analysis. Am. J. Occup. Ther. Off. Publ. Am. Occup. Ther. Assoc..

[25] Beaudry-Bellefeuille I. (2015). Cultural Adaptation for Spain of the Spanish Version of the Short Sensory Profile Using Cognitive Interviews. Austin J. Autism Relat. Disabil..

[26] Guxens M., Ballester F., Espada M., Fernández M.F., Grimalt J.O., Ibarluzea J., Olea N., Rebagliato M., Tardón A., Torrent M. (2012). Cohort Profile: The INMA--INfancia y Medio Ambiente—(Environment and Childhood) Project. Int. J. Epidemiol..

[27] Serra-Majem L., Ribas L., Ngo J., Ortega R.M., García A., Pérez-Rodrigo C., Aranceta J. (2004). Food, Youth and the Mediterranean Diet in Spain. Development of KIDMED, Mediterranean Diet Quality Index in Children and Adolescents. Public Health Nutr..

[28] Tomás Vila M., Miralles Torres A., Beseler Soto B. (2007). Spanish version of the Pediatric Sleep Questionnaire (PSQ). A useful instrument in investigation of sleep disturbances in childhood. Reliability analysis. An. Pediatr. Barc. Spain 2003.

[29] Nolvi S., Merz E.C., Kataja E.-L., Parsons C.E. (2023). Prenatal Stress and the Developing Brain: Postnatal Environments Promoting Resilience. Biol. Psychiatry.

[30] Gouze K.R., Hopkins J., LeBailly S.A., Lavigne J.V. (2009). Re-Examining the Epidemiology of Sensory Regulation Dysfunction and Comorbid Psychopathology. J. Abnorm. Child Psychol..

[31] Gunn T.E., Tavegia B.D., Houskamp B.M., McDonald L.B., Bustrum J.M., Welsh R.K., Mok D.S. (2009). Relationship between Sensory Deficits and Externalizing Behaviors in an Urban, Latino Preschool Population. J. Child Fam. Stud..

[32] Román-Oyola R., Reynolds S. (2013). Prevalence of Sensory Modulation Disorder among Puerto Rican Preschoolers: An Analysis Focused on Socioeconomic Status Variables. Occup. Ther. Int..

[33] Letourneau N.L., Duffett-Leger L., Levac L., Watson B., Young-Morris C. (2013). Socioeconomic Status and Child Development: A Meta-Analysis. J. Emot. Behav. Disord..

[34] Jednoróg K., Altarelli I., Monzalvo K., Fluss J., Dubois J., Billard C., Dehaene-Lambertz G., Ramus F. (2012). The Influence of Socioeconomic Status on Children’s Brain Structure. PLoS ONE.

[35] Vukojević M., Zovko A., Talić I., Tanović M., Rešić B., Vrdoljak I., Splavski B. (2017). Parental Socioeconomic Status as a Predictor of Physical and Mental Health Outcomes in Children—Literature Review. Acta Clin. Croat..

[36] Shavers V.L. (2007). Measurement of Socioeconomic Status in Health Disparities Research. J. Natl. Med. Assoc..

